# Pathway-Based Drug Response Prediction Using Similarity Identification in Gene Expression

**DOI:** 10.3389/fgene.2020.01016

**Published:** 2020-09-09

**Authors:** Seyed Ali Madani Tonekaboni, Gangesh Beri, Benjamin Haibe-Kains

**Affiliations:** ^1^Princess Margaret Cancer Centre, Toronto, ON, Canada; ^2^Department of Medical Biophysics, University of Toronto, Toronto, ON, Canada; ^3^Department of Computer Science, University of Toronto, Toronto, ON, Canada; ^4^Ontario Institute of Cancer Research, Toronto, ON, Canada; ^5^Vector Institute, Toronto, ON, Canada

**Keywords:** breast cancer, human epidermal growth factor receptor 2, lapatinib, trastuzumab, transcriptional similarity coefficient, estrogen receptor

## Abstract

Lapatinib and trastuzumab (Herceptin) are targeted therapies designed for patients with HER2+ breast tumors. Although these therapies improved survival rates of patients with this tumor type, not all the patients harboring HER2 amplification respond to these drugs. The NeoALTTO clinical trial was designed to test whether a higher response rate can be achieved by combining lapatinib and trastuzumab. Although the combination therapy showed almost double the response rate compared to the monotherapies, 40% of the patients did not respond to the treatment. In this study, we sought to identify biomarkers of HER2+ breast cancer patients’ response to drugs relying on gene expression profiles of tumors. We show that univariate gene expression-based biomarkers are significant but weak predictors of drug response. We further show that pathway activities, estimated from gene expression patterns quantified using the recent transcriptional similarity coefficient (TSC) between the tumor samples, yield high predictive value for therapy response (concordance index >0.8, *p* < 0.05). Moreover, machine learning models, built using multiple algorithms including logistic regression, naive Bayes, random forest, k-nearest neighbor, and support vector machine, for predicting drug response in the NeoALTTO clinical trial, resulted in lower performance compared to our pathway-based approach. Our results indicate that transcriptional similarity of biological pathways can be used to predict lapatinib and trastuzumab response in HER2+ breast cancer.

## Introduction

Unsupervised clustering of breast tumor samples based on high-throughput expression profiles enabled the identification of HER2+ breast cancer subtype (Carey et al., 2006; Wirapati et al., 2008; Onitilo et al., 2009). Breast cancer survival differed by subtype (*p* < 0.001), with shortest survival among HER2+ and basal-like subtypes. To treat HER2+ tumors, trastuzumab and lapatinib have been designed to target the EGFR/ERBB2 pathway and yielded a 30% rate of clinical response in the NeoALTTO clinical trial for patients with HER2+ breast tumors (Baselga et al., 2012). This led to their adoption as standard-of-care therapies for HER2+ breast cancer patients (Baselga et al., 2012). To increase the response rate of HER2+ tumors, the biomedical research community sought to design combination therapies. Concurrent treatment with these drugs with a response rate of almost 60% proved a higher efficacy of combination therapy with respect to lapatinib and trastuzumab as monotherapies (Baselga et al., 2012). However, there is a need to identify the non-responders to further improve the rate of treatment response in HER2+ breast cancer patients.

Stratification of responders and non-responders to lapatinib, trastuzumab, and their combination therapies was conducted using either gene-based or pathway (or gene set)-based approaches. In gene-based approaches, mutation, amplification, or expression of individual genes with known association with HER2+ breast tumor biology was investigated as potential biomarkers of response to these therapies (Gomez et al., 2007; Bianchini et al., 2011; Dave et al., 2011; Loibl et al., 2014; Schneeweiss et al., 2014; Vici et al., 2014; Menyhárt et al., 2015). In spite of successful studies as part of gene-based analysis, they could not capture the whole picture of resistance mechanisms to targeted therapies in HER2+ breast cancer patients due to the complexity of HER2+ tumor biology (Nahta, 2012; de Melo Gagliato et al., 2016; Zhang et al., 2017). Hence, pathway (or gene set) approaches were conducted trying to identify the association of multiple genes or pathways to therapy response in HER2+ breast cancer patients.

The association of biological pathways to treatment response in HER2+ breast cancer patients has been conducted based on pathway enrichment analysis using individual genes identified either (1) based on higher activity in responders (or non-responders) versus non-responders (or responders) (Wu et al., 2012; Boulbes et al., 2015; Nam et al., 2015) or (2) as individual or multigene biomarkers of response (Harris et al., 2007; Willis et al., 2018). Here we present an alternative approach where pathways are used directly as features for predicting treatment response in HER2+ breast cancer patients.

In this study, we used our recent Similarity Identification in Gene Expression (SIGN) approach (Madani Tonekaboni et al., 2019) as a classifier relying on expression patterns in biological pathways for the classification of patient tumor samples to predict the response of cancer patients in each arm of the NeoALTTO clinical trial. We showed that the transcriptional similarity coefficient (TSC), identified comparing each patient tumor sample to responders versus non-responders, can be used to identify new pathway-based biomarkers of drug response in HER2+ breast cancer patients.

## Materials and Methods

**FIGURE 1:**
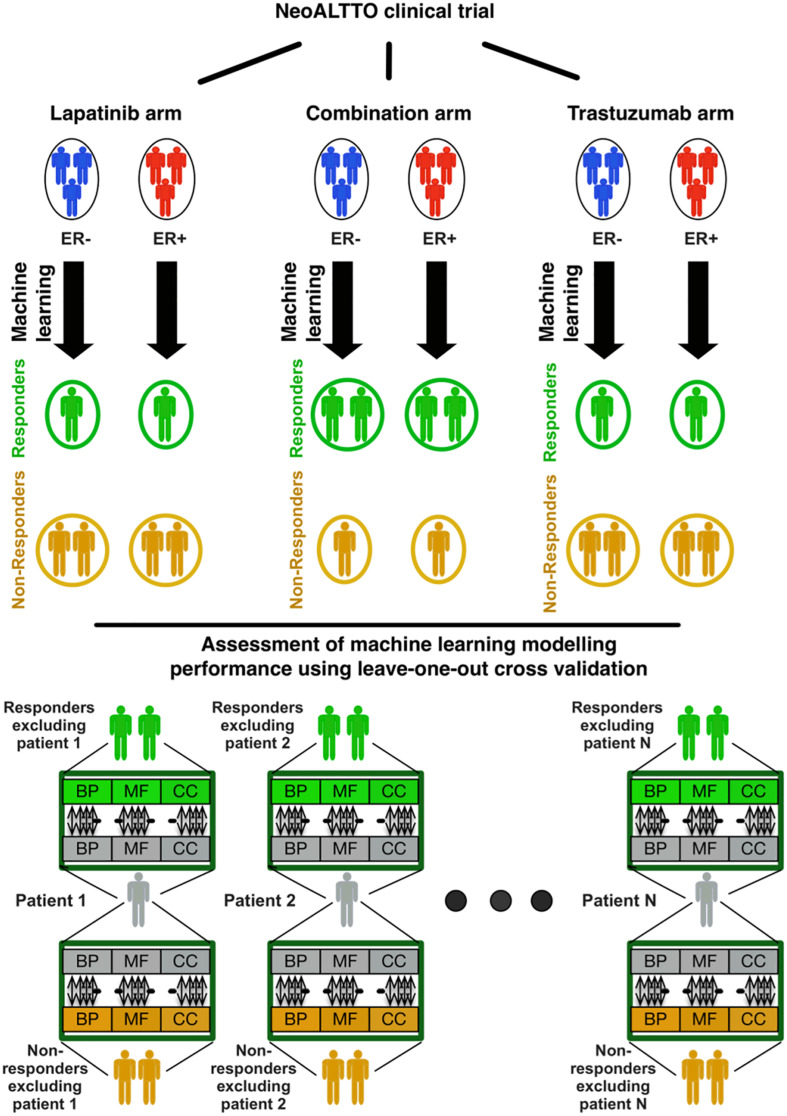
Design of the study regarding identification of responders to lapatinib, trastuzumab, and their combination therapy in the NeoALTTO clinical trial. BP, MF, and CC stand for the Gene Ontology (GO) terms for biological processes, molecular functions, and cellular components, respectively.

The overall design of our study is illustrated in Figure 1. In brief, we used the similarity of patterns of gene expression in biological pathways from patients responding to lapatinib, trastuzumab, and their combination to predict therapy response using our SIGN methodology (Madani Tonekaboni et al., 2019). In this framework, a leave-one-out cross-validation was used to assess the performance of each biomarker in predicting the response of cancer patients in each treatment category (Figure 1). The data and detailed methods are described below.

### Gene Expression Profiles of Tumor Samples

RNA-seq raw data of tumor samples in the NeoALTTO clinical trial were quantified with Kallisto (Bray et al., 2016) in Toil pipeline (Vivian et al., 2017) using the GENCODE version 23 (ALL version) transcriptome annotation. Transcript level abundances are summarized to gene level using the same approach as described in Soneson et al. (2015).

### Clinical Definition of Responders Versus Non-responders

Responders and non-responders in the NeoALTTO clinical trial were determined using the rate of pathological complete response (pCR) (Baselga et al., 2012). Any patient without a recorded pCR was regarded as a non-responder. A pathological complete response is defined as no invasive cancer in the breast or only non-invasive *in situ* cancer in the breast specimen. Surgical breast and axillary node resection specimens were evaluated for pathologic tumor response according to the National Surgical Adjuvant Breast and Bowel Project (NSABP) guidelines^1^.

### Unsupervised Clustering

Similarities of samples within each arm of the NeoALTTO clinical trial were identified using Spearman’s rank-order correlation (Wissler, 1905). The hierarchical clustering was then implemented on the similarity matrix between the samples using Euclidean distance and Ward’s minimum variance method (Murtagh and Legendre, 2014).

### Univariate Biomarker Discovery Using Genes

Concordance indices between the expression of each gene and the binarized vector of drug response were calculated as the prediction performance of each gene as a univariate biomarker. The significance of each identified C-index was calculated using a permutation test. The observations were randomly permuted, and the C-index between the expression of each gene and the observed classes for the tumor samples was calculated. Then the fraction of times in which the C-index of the gene expression with real observed classes was lower than the C-indices identified with permuted observed classes was considered as the significance (or FDR) of the C-index identified for that gene.

### Concordance Index

We used the concordance index (C-index) to quantify the predictive value of our drug response predictors. The C-index estimates the probability that, for a pair of randomly chosen comparable samples, the sample with the higher predicted value will experience an event before the other sample or belongs to a higher binary class (Harrell et al., 1982). We used the implementation of the concordance index available in the survcomp R package (version 1.34.0) (Schröder et al., 2011).

### Transcriptional Similarity Coefficient

The transcriptional similarity coefficient (TSC) between each sample and the responders and non-responders were identified using the TSC function in the SIGN R package (version 0.1.0) (Madani Tonekaboni et al., 2019). Let *P* be the matrix of expression of genes within a pathway for a set of biological samples where rows are genes and columns are samples. Then the TSC is defined as follows:

$$T\,S\,C\,\left(P_{1}\,P_{2}\right)=\frac{\sum_{i}{\left(P_{10}\times P_{20}\right)}_{i\,i}}{\sqrt{\sqrt{\sum_{i\,j}{\left(P_{10}\right)}_{i\,j}^{2}}\,\sqrt{\sum_{i\,j}{\left(P_{20}\right)}_{i\,j}^{2}}}}$$

where *P*_1_ and *P*_2_ represent the matrix of gene expressions of a given pathway in two sets of samples (populations 1 and 2), *i* is the row index (i.e., gene index) within each matrix, *j* is the column index (i.e., sample index) within each matrix, and *P*_m__0_ (either *P*_10_ or *P*_20_)

$$P_{m\,0}=P_{m}\times P_{m}^{\prime }-D\,i\,a\,g\,o\,n\,a\,l\,\left(P_{m}\times P_{m}^{\prime }\right)$$

where *m* is either 1 for population 1 or 2 for population 2. Deducting the diagonal elements in the above equation was initially proposed to the bioinformatics community for analyzing genomics data (Smilde et al., 2009). This term will make sure that the identified similarities do not depend on the number of samples compared between the datasets.

The TSC captures the similarity of the pathway expression pattern between two samples and/or sample sets that is in the range [−1,1].

### Identifying Responders Using TSC

The TSC for each pathway was identified between one sample and the remaining samples, divided into two groups of responders and non-responders (Baselga et al., 2012). GO terms in level C5 with 10 to 30 genes are used in this study to identify the similarity between samples based on their gene expression pattern (Madani Tonekaboni et al., 2019). We limited the number of genes in GO terms to exclude large GO terms (at the top of the GO term hierarchy) that are parents of the GO terms in our study (at the bottom of the GO hierarchy). If the TSC for similarity for the responders was higher than that for the non-responders, the given sample was considered as a responder and *vice versa*. This process was repeated for every given sample in each arm of the trial. The method’s performance for predicting the response of cancer patients was assessed using the concordance index.

### Cross-Validation in Predictive Models

Each model was validated using leave-one-out cross-validation. In this setting, a target sample was put aside, and the rest of the samples were used for the prediction of drug response in the target sample. The TSC of each pathway between the set-aside sample and the randomly selected five samples from responders and non-responders were calculated. Then the median of the TSCs of all the pathways was calculated to assess if the sample has a higher similarity to responders or non-responders. This process was repeated 100 times for each sample, and majority votes of the 100 times were considered as the predicted class of the sample to be responder or non-responder.

## Results

We leveraged the gene expression and clinical information of HER2+ breast cancer patients in the NeoALTTO clinical trial to identify biomarkers of drug response. The NeoALTTO clinical trial was a phase three randomized clinical trial designed to assess the efficacy of anti-HER2 monoclonal antibody trastuzumab, the tyrosine kinase inhibitor lapatinib, and their combination therapies on HER2-overexpressing breast cancer patients. The response (pCR) rate was significantly higher in the group given lapatinib and trastuzumab (51.3%) than in the group given trastuzumab alone (29.5%; *p* < 0.05). However, no significant difference in pCR between the lapatinib (24.7%) and the trastuzumab (*p* = 0.34) groups was observed.

**FIGURE 2:**
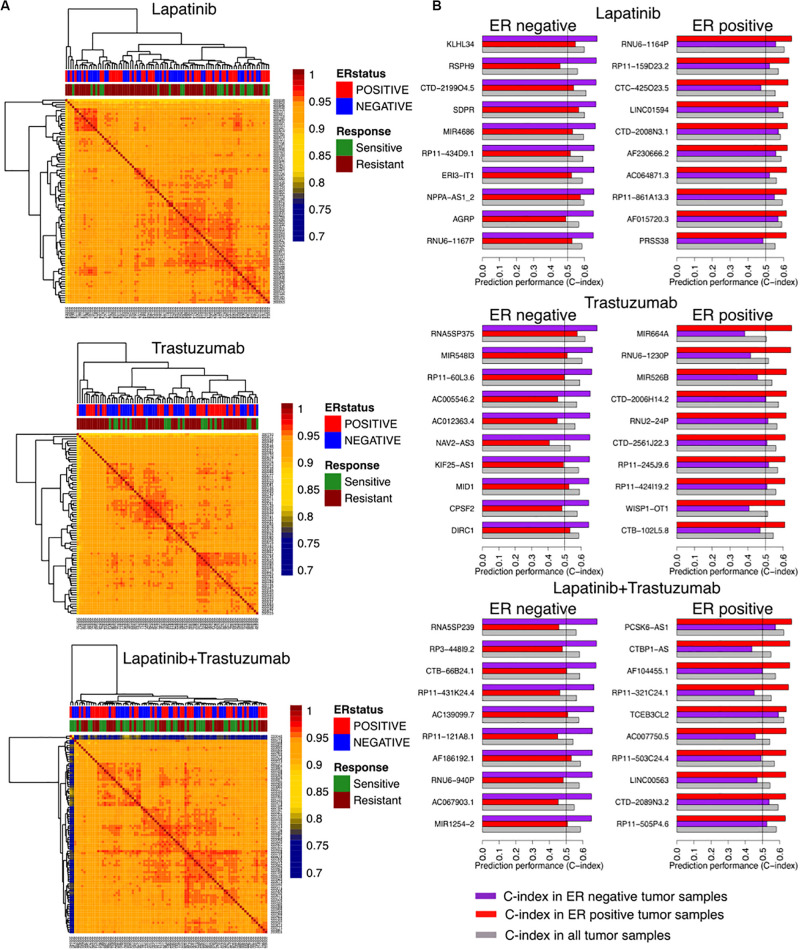
Identifying responders to lapatinib, trastuzumab, and their combination therapy in the NeoALTTO clinical trial using genes as univariate predictors of response. **(A)** Clustering of samples based on their similarity, defined as the Spearman correlation between gene expression profiles of the sample. **(B)** Top 10 genes as univariate biomarkers of drug response in ER+ and ER– cohorts within each arm of the NeoALTTO clinical trial.

We identified correlations of tumor samples based on their gene expression profiles in three arms of the clinical trial separated based on the treatment type including trastuzumab alone, lapatinib alone, and their combination therapies (Figure 1). The unsupervised clustering of samples could not stratify the patients based on their responses, relying on the rate of pathological complete response (Figure 2A).

We further computed the C-index of genes as univariate biomarkers of drug response in each arm of the NeoALTTO trial. Relying on the common knowledge on ER being one of the main drivers in breast cancer development and progression (Fuqua, 1997), we stratified our analyses based on the ER status. Top predictors of response yield a C-index of 0.68 (Figure 2B), while the C-index of ERBB2 as a univariate biomarker of response in all the arms does not exceed 0.59 (the full list can be found in the Supplementary Material). The low performance of univariate modeling could be due to high correlation of patient tumor samples, as more than 90% of the tumor sample pairs had Pearson correlation of more than 0.9 using their gene expression profiles.

**FIGURE 3:**
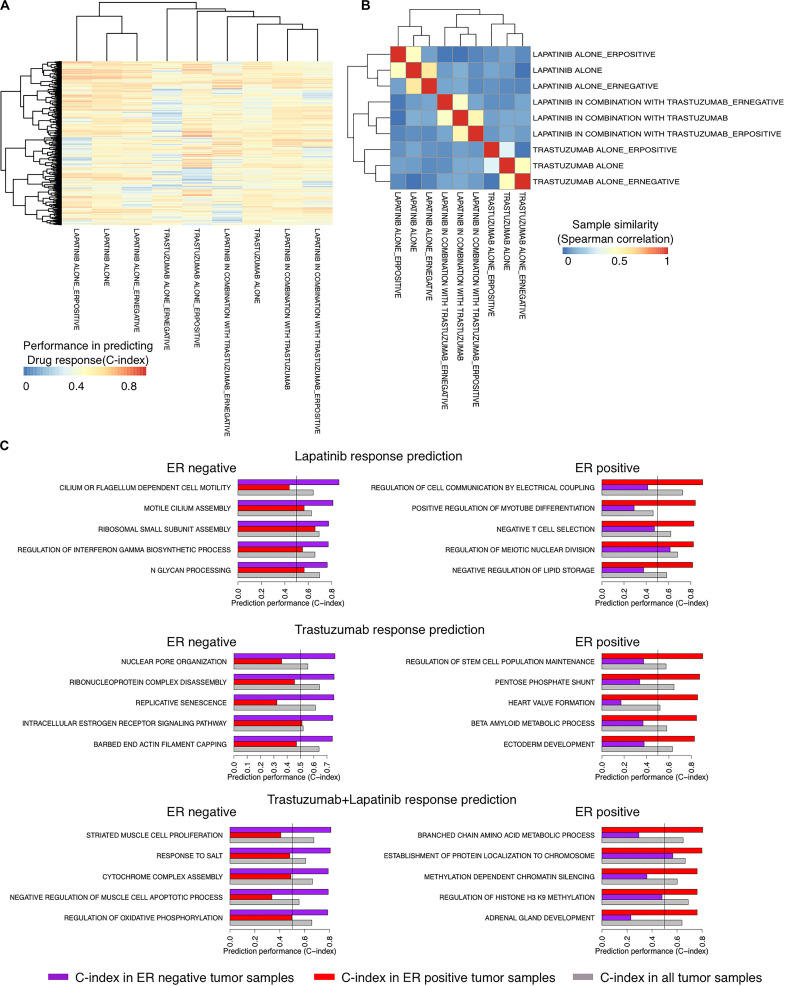
Identifying responders to lapatinib, trastuzumab, and their combination therapy in the NeoALTTO clinical trial using the transcriptional similarity coefficient (TSC) of pathways. **(A)** Concordance indices of delta TSCs of GO terms, comparing each sample with responders and non-responders, in predicting the response of patients to lapatinib, trastuzumab, and their combination. **(B)** Clustering of groups of patients based on Concordance indices of delta TSC of GO terms **(A)**. **(C)** Top pathways as predictors of lapatinib, trastuzumab, and their combination in ER+ and ER– tumor samples in the NeoALTTO clinical trial.

We recently showed the high performance of a new method called SIGN in predicting the survival rate of breast cancer patients under different therapeutic regimens (Madani Tonekaboni et al., 2019). We sought to use SIGN to predict the drug response of patients in each arm of the NeoALTTO trial. We used C-indices of the pathways, identified between the TSC of the pathways and the drug response in each arm of the trial, to cluster the arms. Trastuzumab alone and the combination therapy arms were clustered more closely compared to the lapatinib alone arm using the C-indices of the pathways, although the difference is not significant (*p* > 0.05) (Figure 3A). Moreover, the pathway biomarkers of ER− and ER+ patient tumors showed low commonality revealing differences in the mechanism of response caused by the ER status of the patient tumors (absolute Spearman correlation <0.08) (Figure 3B). Top pathway biomarkers for patients with the same treatment regimen and ER status had C-indices of more than 0.8 except for ER− patients under trastuzumab alone therapy (Figure 3C).

Although the biological function of the identified pathways as biomarkers of drug response requires experimental validation, we found some evidence on their biological relevance. For example, among top identified biomarkers of drug response, there are REGULATION OF INTERFERON GAMMA BIOSYNTHETIC PROCESS and NEGATIVE T-CELL SELECTION for ER− and ER+ cancer patients under lapatinib treatment, respectively. These are in agreement with previous literature on the importance of immune signaling in lapatinib response in cancer patients (Griguolo et al., 2019).

### Comparison With Other Machine Learning Models

**FIGURE 4:**
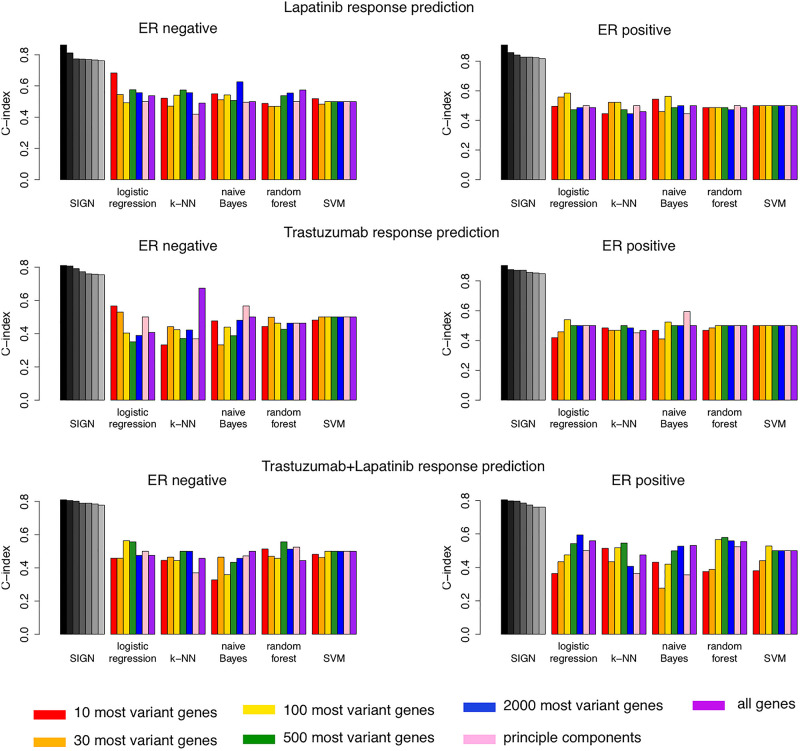
Comparison of performance of top seven biomarkers of drug response using Similarity Identification in Gene Expression (SIGN) and 35 machine learning models built combining five machine learning methods and seven different feature selection approaches.

We compared the top seven biomarkers identified in each arm of the NeoALTTO clinical trial for patients with ER− or ER+ status with the performance of 35 machine learning models (Figure 4). These models were built using five different machine learning algorithms, including logistic regression, k-nearest-neighbor (k-NN), naive Bayes, random forest, and support vector machine (SVM), and seven different feature selection approaches (Figure 4). SIGN-based biomarkers outperformed all 35 models in all treatment categories. We used the same leave-one-out cross-validation strategy as used for SIGN to compare the performance of these models.

## Discussion

We propose SIGN as a new approach to identify biomarkers of drug response in other subtypes of breast cancer or other tumor types. We showed the utility of SIGN in predicting the response of HER2+ breast cancer patients to lapatinib, trastuzumab, and their combination therapies using transcription patterns within biological pathways. Our results further emphasize the information gained upon using genes within biological pathways instead of individual markers of drug response. Furthermore, it suggests transcriptional similarity coefficient (TSC) as a new measure of similarity between tumor samples to be used in predicting their response to drug response. SIGN-based biomarkers outperformed 35 different machine learning models in predicting drug response in each treatment category. Moreover, the SIGN approach provides us with highly interpretable pathway-based biomarkers of drug response. Although SIGN showed promising performance for predicting response to lapatinib, trastuzumab, and their combination in HER2+ breast cancer patients, this approach needs further validation to ensure its generalizability in new clinical datasets. Upon having access to further clinical data of HER2+ patients in each one of these treatment categories, our findings in this study can be further assessed and validated.

## Data Availability Statement

The datasets generated for this study can be found in the ClinicalTrials.gov Identifier: NCT00553358.

## Author Contributions

SM led the project and performed the computational analysis of the work under supervision of BH-K. GB and SM collected and curated the data. All authors contributed to the article and approved the submitted version.

## Conflict of Interest

The authors declare that the research was conducted in the absence of any commercial or financial relationships that could be construed as a potential conflict of interest.

## Footnotes

1 https://clinicaltrials.gov/ct2/show/results/NCT00553358
